# Patterning mechanisms diversify neuroepithelial domains in the *Drosophila* optic placode

**DOI:** 10.1371/journal.pgen.1007353

**Published:** 2018-04-20

**Authors:** Abhishek Kumar Mishra, F. Javier Bernardo-Garcia, Cornelia Fritsch, Tim-Henning Humberg, Boris Egger, Simon G. Sprecher

**Affiliations:** Department of Biology, University of Fribourg, Fribourg, Switzerland; Indiana University, UNITED STATES

## Abstract

The central nervous system develops from monolayered neuroepithelial sheets. In a first step patterning mechanisms subdivide the seemingly uniform epithelia into domains allowing an increase of neuronal diversity in a tightly controlled spatial and temporal manner. In *Drosophila*, neuroepithelial patterning of the embryonic optic placode gives rise to the larval eye primordium, consisting of two photoreceptor (PR) precursor types (primary and secondary), as well as the optic lobe primordium, which during larval and pupal stages develops into the prominent optic ganglia. Here, we characterize a genetic network that regulates the balance between larval eye and optic lobe precursors, as well as between primary and secondary PR precursors. In a first step the proneural factor Atonal (Ato) specifies larval eye precursors, while the orphan nuclear receptor Tailless (Tll) is crucial for the specification of optic lobe precursors. The Hedgehog and Notch signaling pathways act upstream of Ato and Tll to coordinate neural precursor specification in a timely manner. The correct spatial placement of the boundary between Ato and Tll in turn is required to control the precise number of primary and secondary PR precursors. In a second step, Notch signaling also controls a binary cell fate decision, thus, acts at the top of a cascade of transcription factor interactions to define PR subtype identity. Our model serves as an example of how combinatorial action of cell extrinsic and cell intrinsic factors control neural tissue patterning.

## Author summary

Genetic mechanisms patterning neuroepithelial domains are a critical first step to diversify cellular identity of the developing nervous system. How many cells develop from distinct neuroepithelial domains depends on overall size and growth but also on positioning of boundaries. Using the embryonic optic placode of the fruit fly *Drosophila melanogaster* as a model, we identify basic genetic mechanisms of how distinct domains with different fates emerge from an early, seemingly uniform, neurogenic region. We show that the boundary between two transcription factors is critical to determine how many cells are incorporated in either domain. This is achieved by coordinated interaction of Hedgehog and Notch signaling, which control proliferation and regulate domain-specific transcription factors. The mechanisms employed here in an epithelial placode to determine photoreceptor precursors display similarities with the ones previously identified in the adult compound eye, further supporting the notion of a common developmental program for the larval eye and adult compound eye.

## Introduction

In the fruit fly *Drosophila melanogaster*, all parts of the visual system develop from an optic placode, which forms in the dorsolateral region of the embryonic head ectoderm [1–3]. During embryogenesis, neuroepithelial cells of the optic placode are patterned to form two subdomains. The ventroposterior domain gives rise to the primordium of the larval eye and consists of two photoreceptor (PR) precursor types (primary and secondary precursors), whereas the dorsal domain harbors neuroepithelial precursors that generate the optic lobe of the adult visual system [4–6]. The basic helix-loop-helix transcription factor Atonal (Ato) promotes PR precursor cell fate in the larval eye primordium [4,7]. The orphan nuclear receptor Tailless (Tll) is confined to the optic lobe primordium and maintains non-PR cell fate [4]. Hedgehog (Hh) and Notch (N) signaling are critical during the early phase of optic lobe patterning. The secreted Hh protein is required for the specification of various neuronal and non-neuronal cell types, while Notch acts as neurogenic factor preventing ectodermal cells from becoming neuronal precursors by a process termed lateral inhibition [8,9]. In the optic placode Ato expression is promoted by Hh and the retinal determination genes *sine oculis* (*so*) and *eyes absent* (*eya*). Notch delimits the number of PR precursors and maintains a pool of non-PR precursors [10]. Ato is initially expressed in all PR precursors in the placode and its expression gets progressively restricted to primary precursors [7,11]. In a second step, primary precursors recruit secondary precursors via EGFR signaling: primary precursors express the EGFR ligand Spitz, which is required in secondary precursors to promote their survival. After this initial specification of primary and secondary PR precursors, the transcription factors Senseless (Sens), Spalt (Sal), Seven-up (Svp) and Orthodenticle (Otd) coordinate PR subtype specification. Sens and Spalt are expressed in primary PR precursors, while Svp contributes to the differentiation of secondary PR precursors [12,13]. By the end of embryogenesis, primary PR precursors have fully differentiated into blue-tuned Rhodopsin5 PRs (Rh5), while secondary PR precursors have differentiated into green-tuned Rhodopsin6 PRs (Rh6) [12,13]. While the functional genetic interactions of transcription factors controlling PR subtype specification has been thoroughly studied, it remains unknown how the placode is initially patterned by the interplay of Hh and Notch signaling pathways. Similarly, the mechanisms of how *ato* and *tll*-expressing domains are set up to ensure the correct number of primary and secondary PR precursors as well as non-PR precursors of the optic lobe primordium remain unknown.

Here we describe the genetic mechanism of neuroepithelial patterning and acquisition of PR versus non-PR cell fate in the embryonic optic placode and provide the link to subsequent PR subtype identity specification. The non-overlapping expression patterns of *ato* and *tll* in the optic placode specifically mark domains giving rise to the larval eye precursors (marked by Ato) and the optic lobe primordium (marked by Tll). *ato* expression in the larval eye primordium is temporally dynamic and can be subdivided into an early *ato* expression domain, including all presumptive PR precursors and a late *ato* domain, restricted to presumptive primary PR precursors. The *ato* expression domain directly forms a boundary adjacent to *tll* expressing precursors of the optic lobe primordium. We show that *tll* is both necessary and sufficient to delimit primary PR precursors by regulating *ato* expression. Hh signaling regulates the cell number in the optic placode and controls PR subtype specification in an *ato*- and *sens*-dependent manner. Finally, we also show that Notch has two temporally distinct roles in larval eye development. Initially, Notch represses *ato* expression by promoting *tll* expression and later, Notch controls the binary cell fate decision of primary versus secondary PR precursors by repressing *sens* expression. In summary, we identify a network of genetic interactions between cell-intrinsic and cell-extrinsic developmental cues patterning neuroepithelial cells of the optic placode and ensuring the timely specification of neuronal subtypes during development.

## Results

### Expression patterns of *atonal* and *tailless* subdivide the optic placode

During embryonic development, the *Drosophila* optic placode produces both the larval eye PRs and the precursors of the optic lobe [14]. To document how the boundary between these two groups of cells is established, we mapped the expression patterns of a subset of proteins that are expressed in different subregions within the optic placode.

**Fig 1:**
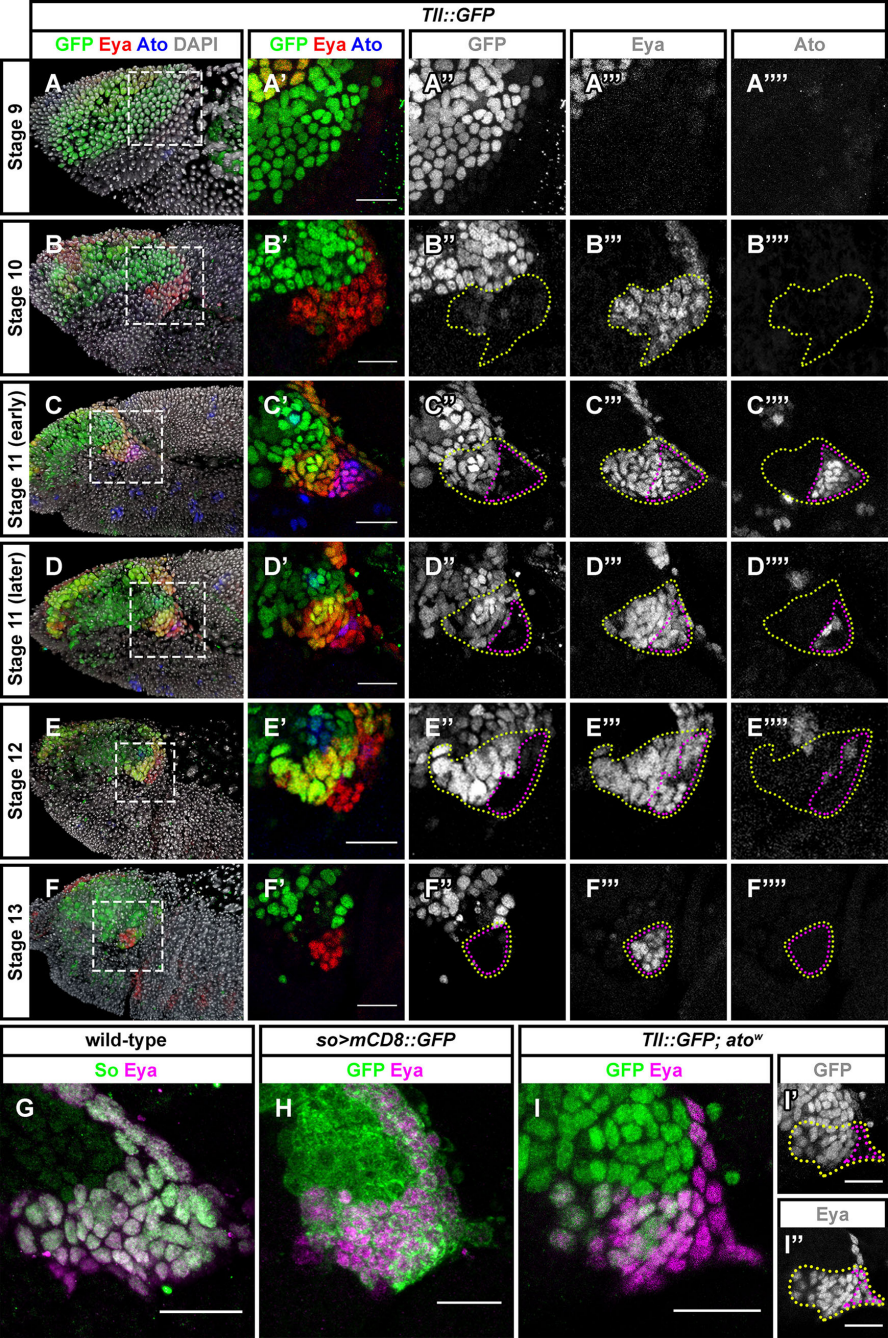
The Eya-positive domain of the optic placode is subdivided at stage 11. (A-F) Eya (red), Tll-GFP (green) and Ato (blue) expression patterns in the embryonic optic placode at stages 9–13. At stage 10 a patch of Eya-positive cells is detected within the ventral most region of the optic placode (B, outlined in yellow). Some of these cells start expressing Ato during stage 11 and will form the Bolwig's organ (C, outlined in magenta), whereas other cells expressing Tll will form the optic neuropile. Ato expression is progressively restricted during stages 11 and 12 (D, E) and is no longer detectable at stage 13 (F). The retinal determination network transcription factor So is co-expressed with Eya in the optic placode (G, H). *ato* is not required for restricting Tll expression (I). Scale bars represent 20 μm.

The optic placode is first detected on the surface of embryos at stage 10, located in the posterior procephalic region. During stage 10, the transcription co-activator Eya starts being expressed in a crescent-shaped domain, overlapping with the ventral-most region of the optic placode (Fig 1A and 1B) [11]. At this stage, virtually all Eya-positive cells within the optic placode display co-staining with an antibody against phospho-Histone H3 (pH3), a mitotic marker, indicating that these cells divide during stage 10 (S1 Fig). At stage 11, a boundary appears within the optic placode that subdivides the Eya-expressing domain. Cells that are located anteriorly start expressing the nuclear receptor Tll, and cells located posteriorly start expressing the basic helix-loop-helix transcription factor Ato. Ato is required for the development of the Bolwig's organ, whereas Tll is important for the formation of the optic lobe. Tll expression is maintained later in development, during stages 12 and 13, whereas Ato expression is lost progressively during these stages (Fig 1C–1F). Both Eya and Sine oculis (So), which are components of the retinal determination network and, therefore, important for adult eye development [15], are co-expressed in the optic placode (Fig 1G and 1H).

It has been suggested that the pool of late Ato-expressing cells will give rise to the primary PR precursors [11,16]. Tll represses Ato in the optic placode, and the number of Bolwig's organ PRs is increased in *tll* mutants. To see if Ato is responsible for repressing *tll*, we looked at the expression of the Tll::GFP reporter in *ato* mutants and found no spreading of Tll::GFP expression into the posterior Eya-positive cells (Fig 1I). Hence, Ato is not required to restrict *tll* expression.

### *tll* controls the balance between larval eye precursors and optic lobe neuroepithelium

**Fig 2:**
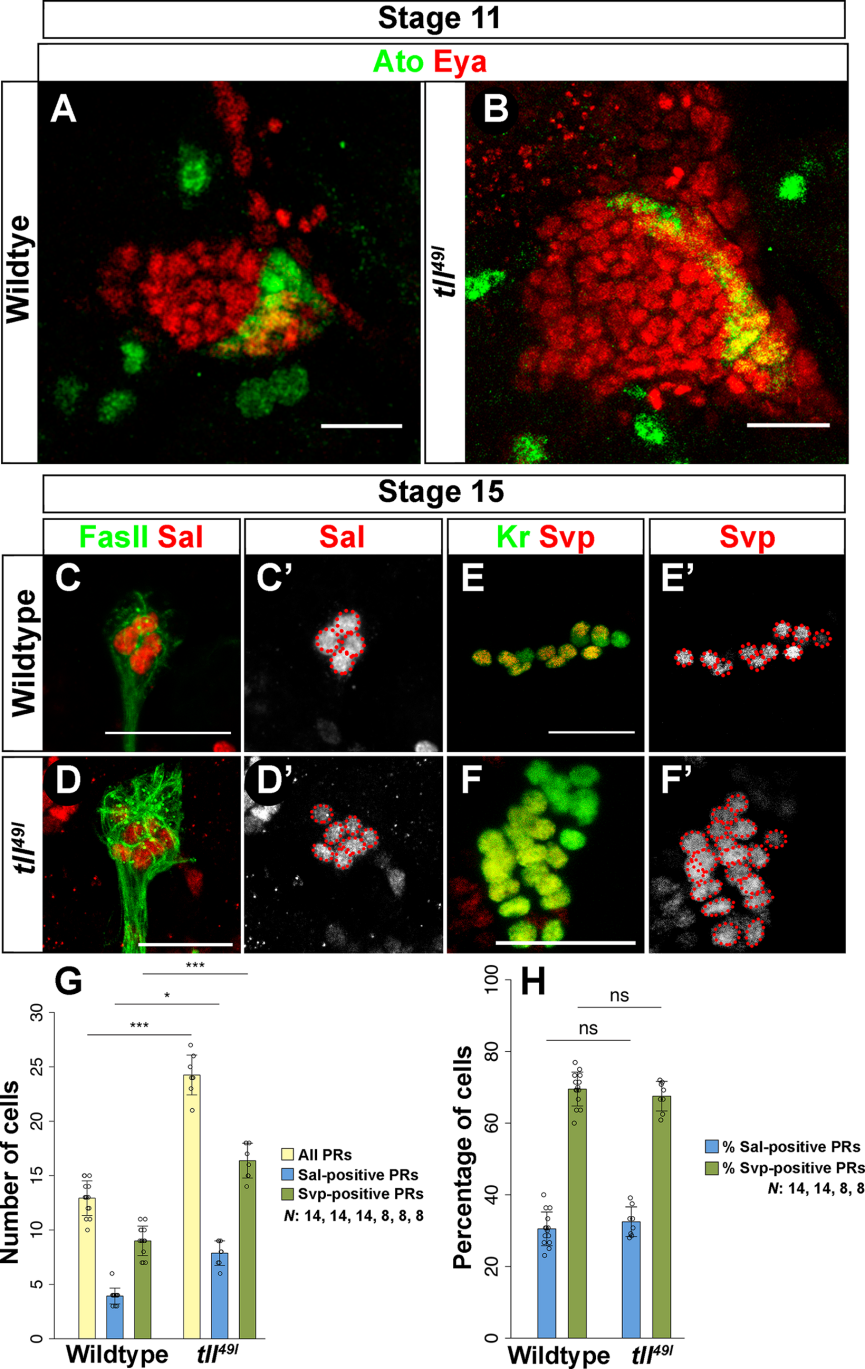
*tll* regulates Ato-dependent PR precursor cell fate in the embryo. (A, B) Ato (green) and Eya (red) expression in the optic placode in wildtype and *tll*^*49l*^ mutants at embryonic stage 11. The *tll* mutant placode is bigger, and, as a consequence, the number of Ato-expressing cells is increased (B). Ato-expression extends within the posterior region of the optic placode. (C, C’, D, D’) Sal (red) expression in wildtype and *tll*^*49l*^ mutant stage 15 embryos. FasII (green) marks differentiated PR neurons in the embryo. The number of Sal-expressing primary precursors is increased in *tll*^*49l*^ mutants (D’) as compared to wildtype (C’). (E, E’, F, F’) Svp (red) expression in wildtype and *tll*^*49l*^ mutant stage 15 embryos. Kr (green) marks PR precursors in the embryo. The number of Svp-expressing secondary precursors is also increased in *tll*^*49l*^ mutants (F’) compared to wildtype (E’). (G, H) All PR numbers (G) and percentages (H) were analyzed in wildtype as well as in *tll* mutants. Also, the cell number and percentage of Sal- and Svp-positive cells in wildtype and *tll*^*49l*^ mutant embryos were quantified, and the ratio of Sal- and Svp-positive PRs is not significantly changed in these two genotypes (G, H). Number of all PRs: Anova: p<0.001 F(5,55) = 92.92; wildtype vs *tll*^*49I*^ p<0.001, t = -4.731; Number of all Sal-positive cells: Anova: p<0.001 F(5,44) = 104.2; wildtype vs *tll*^*49I*^ p = 0.0302, t = -2.851; Number of all Svp-positive cells: Anova: p<0.001 F(5,48) = 70.63; wildtype vs *tll*^*49I*^ p = 0.0009, t = -4.063 (G). Ratio of Sal-positive cells: Anova: p<0.001 F(5,44) = 114.3; wildtype vs *tll*^*49I*^ p = 0.978, t = -0.569; Ratio of Svp-positive cells: Anova: p<0.001 F(5,48) = 59.64; wildtype vs *tll*^*49I*^ p = 0.995, t = 0.402 (H). n = 14 (wildtype), 8 (*tll*^*49I*^) (G, H). Data is shown as mean and error bars as standard deviation. Circles represent numbers or percentages of individual samples. *** p<0.001, *p<0.05 and ns = not significant (G, H). Scale bars represent 20 μm.

As the expression and function of Ato and Tll may be linked, we next focused on the role of Tll in larval optic placode patterning. *tll* is specifically expressed in non-PR precursors in the optic placode [4]. It was previously proposed that Tll counteracts the EGF signal and prevents placode cells from developing as secondary PR precursors [4]. To understand how *tll* regulates subtype specification of larval PR precursors in the optic placode, we first analyzed *ato* expression in *tll* mutants. We found that in *tll* mutants the optic placode possesses about twice as many Eya-positive cells as compared to control embryos, and this increase is paralleled by an increase in the number of Ato-positive cells (Fig 2A and 2B). Interestingly, in *tll* mutants the expression of Ato is not limited to the postero-ventral corner but rather, *ato* expression is expanded and occupies the whole posterior margin of the Eya-positive domain of the optic placode (Fig 2B). Later in development, *tll* mutants form about twice as many PR precursors as control embryos (an average of 24 cells), which is proportional to the increase in placode size. These ectopic PRs express the transcription factor Hazy (S2 Fig), a transcription factor that activates the expression of phototransduction proteins [17, 18], indicating that these cells are correctly specified. We further analyzed the determination of PR-identity by assessing the PR subtype specific markers Spalt (Sal) and Seven-up (Svp). We found an increase in total PR cell number including Sal-expressing primary PR precursors as well as Svp-expressing secondary PR precursors in *tll* mutants (Fig 2C–2G). However, the relative fraction of Sal- or Svp-positive PRs remained unchanged. Thus, the ratio between both PR subtypes in *tll* mutants was comparable to wildtype control (Fig 2H).

**Fig 3:**
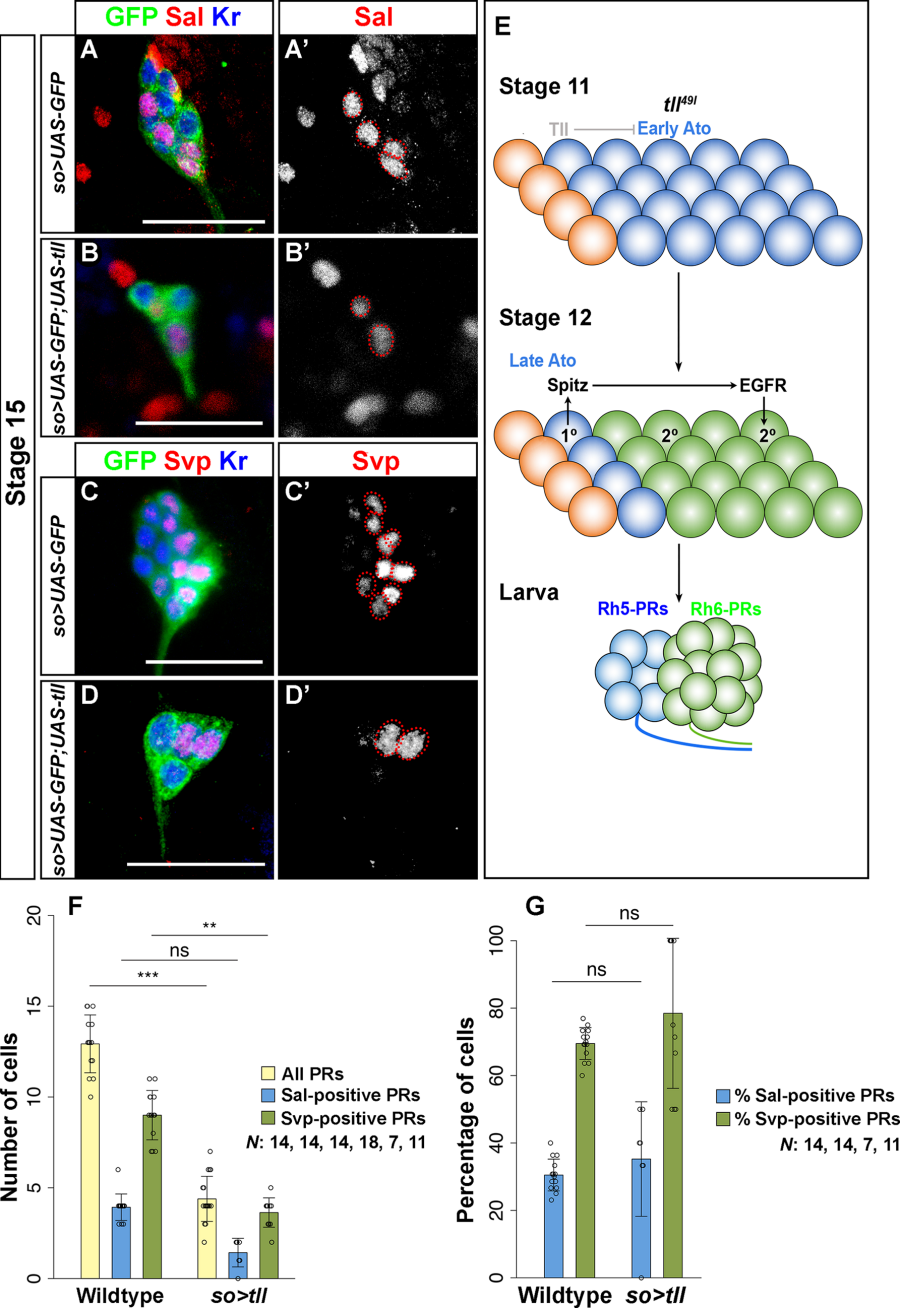
*tll* is required for primary PR precursor cell fate. (A, A’, B, B’) Sal (red) expression in the primary PR precursors in control (*so>UAS-GFP*) and *tll* overexpression (*so>UAS-GFP;UAS-tll*) in embryos at stage 15. GFP (green) marks *so*-expressing cells whereas Kr (blue) marks PR precursors in the embryo. Sal-expressing primary PR precursors are significantly reduced in *tll* overexpressing embryos (B’), indicating that Tll is sufficient for repressing primary PR precursor cell fate in the embryo. (C, C’, D, D’) Svp (red) expression in the secondary PR precursors in control (*so>UAS-GFP*) and *tll*-overexpressing (*so>UAS-GFP;UAS-tll*) embryos at stage 15. Svp-positive secondary PR precursors are also reduced (D’) potentially as a result of the decrease in the number of primary PR precursors (B’); Maximum intensity projections of confocal sections. (E) Schematic representation of Tll mediated neuroepithelial patterning and regulation of PR versus non-PR cell fate in the embryonic optic placode of *tll*^*49l*^ mutants. We show that Tll represses Ato expression and thereby regulates primary PR precursor cell fate. (F, G) We also quantified cell numbers (F) and percentages (G) in *so>tll* embryos. Tll overexpression leads to a reduction in the number of PR precursors that are formed (counted at stages 14–16). Interestingly, *so>tll* animals possess a ratio of Svp- vs Sal-positive PRs which is similar to that of wildtype. Number of all PRs: wildtype vs *so>tll* p<0.001, t = 4.439; Number of all Sal-positive cells: wildtype vs *so>tll* p = 0.3358, t = 1.729; Number of all Svp-positive cells: wildtype vs *so>tll* p = 0.009863, t = 3.250 (F). Ratio of Sal-positive cells: wildtype vs *so>tll* p = 0.612, t = -1.305; Ratio of Svp-positive cells: wildtype vs *so>tll* p = 0.203, t = -2.000 (G). n = 14 (wildtype), 18 (*so>tll;* all PRs), 7 (*so>tll;* Sal-positive cells), 11 (*so>tll;* Svp-positive cells) (F, G). Data is shown as mean and error bars as standard deviation. Circles represent numbers or percentages of individual samples. *** p<0.001, **p<0.01 and ns = not significant (F, G). Scale bars represent 20 μm.

To determine if Tll is also sufficient to genetically repress the PR precursor cell fate, we ectopically expressed Tll under the control of *so-Gal4*. Ectopic expression of Tll does not change the total number of cells in the optic placode, but it does reduce the total number of PRs in the developing larval eye. However, the ratio of Sal-positive and Svp-positive cells remains comparable to that of control embryos (Figs 3A–3G and S3).

These findings suggest that in *tll* mutants, Ato expression expands, and thus, increases the pool of primary PR precursors. As a result, more secondary PR precursors are incorporated into the larval eye (Fig 3E). However, the finding that the ratio of primary and secondary PR precursors remains the same as in wildtype (Fig 2H) suggests that the recruitment of secondary PR precursors by primary PR precursors in *tll* mutants occurs in a normal manner. Taken together, our findings support that Tll acts as a critical cell fate determinant by repressing larval eye precursors (Fig 3E).

### Hedgehog signaling controls the cell number of PR precursors in the optic placode

Hedgehog (Hh) signaling is required for the formation of photoreceptor precursors in the *Drosophila* compound eye, in the ocelli, and in the larval eye. Hh regulates the onset of PR formation in an Ato-dependent manner [6,19, 20]. The canonical Hh signaling pathway includes two transmembrane proteins Patched (Ptc) and Smoothened (Smo) [21]. In the absence of Hh, Ptc represses Smo preventing signal transduction. However, binding of Hh to Ptc eliminates Ptc-dependent repression of Smo activating a downstream signaling cascade [22]. Therefore, *ptc* and *smo* mutant phenotypes correlate with Hh gain- and loss-of-function mutants, respectively. Loss of Hh results in a complete loss of ato expression and thus PR precursor formation, while increased Hh signaling in *ptc* mutants shows an increase in PR cell numbers [6]. Importantly, in *ptc* mutants, these additional PRs express the PR marker Hazy, and, by the end of their development, at stage 17, they further terminally differentiate correctly to express Rh5 or Rh6 (S2 and S4 Figs).

**Fig 4:**
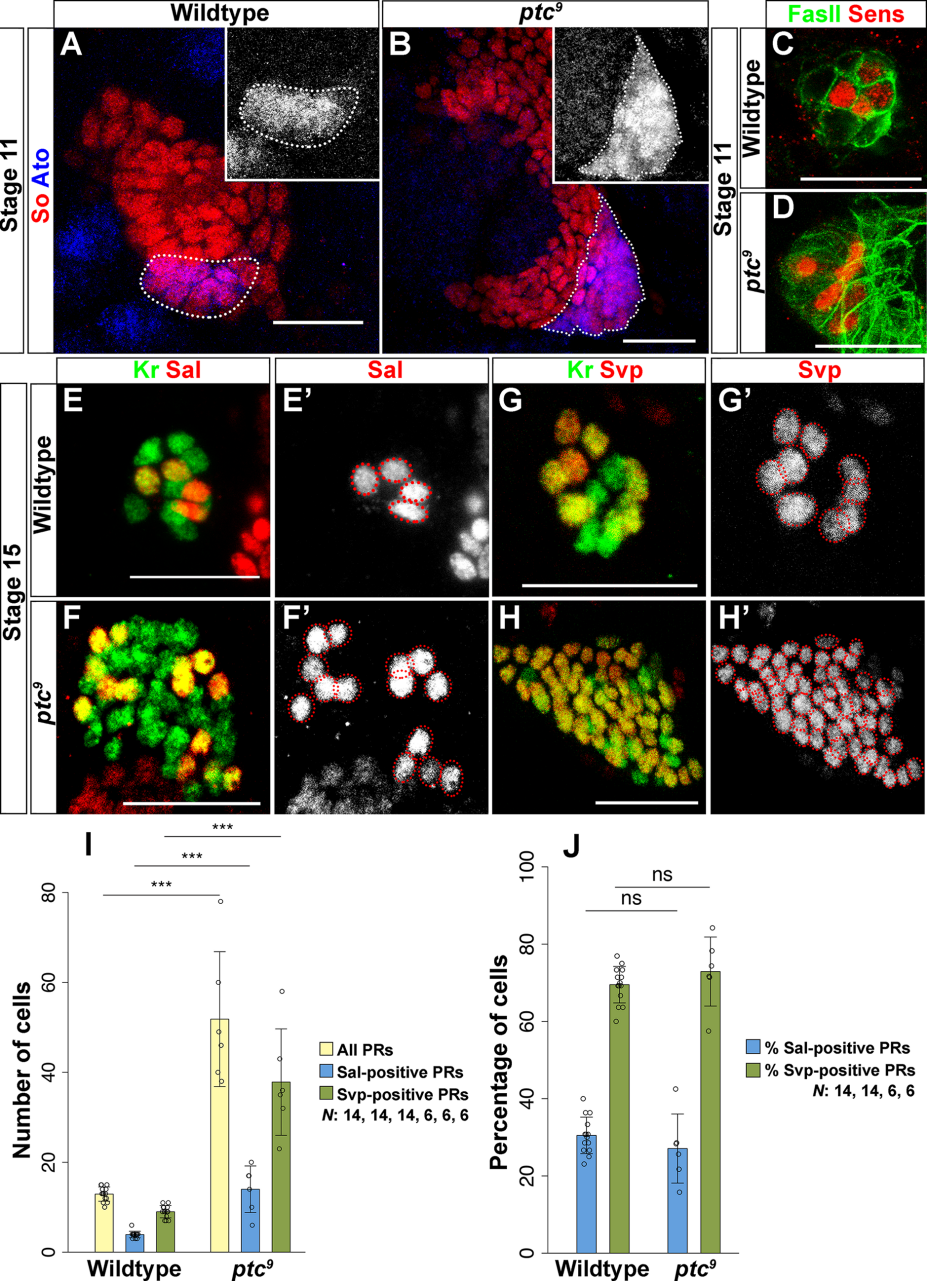
Hh regulates Ato- and Sens-dependent PR cell fate in the embryo. (A, B) Ato (blue) expression in the optic placode in wildtype and *ptc*^*9*^ mutant stage 11 embryos. So (red) marks cells of the entire optic placode at this stage. The number of Ato-expressing cells is increased in *ptc*^*9*^ mutants (B) as compared to wildtype (A). (C, D) Sens (red) expression in the PR precursors of wildtype and *ptc*^*9*^ mutant stage 12 embryos. FasII (green) marks differentiated PR neurons at this stage. The number of Sens expressing cells is also increased in *ptc*^*9*^ mutants (D). (E, E’, F, F’) Sal (red) expression in the PR precursors in wildtype and *ptc*^*9*^ mutant stage 15 embryos. Kr (green) marks PR precursors at this stage. The number of Sal-expressing primary PR precursors is significantly increased in *ptc*^*9*^ mutants (F’) compared to wildtype (E’). (G, G’, H, H’) Svp (red) expression in the PR precursors in wildtype and *ptc*^*9*^ mutant stage 15 embryos. The number of Svp expressing secondary PR precursors is also increased (H’), probably as a result in the increase of primary PR precursors (F’). (I, J) Quantification of PR cell number (I) and percentage of Sal- and Svp-positive PRs in the wildtype and *ptc*^*9*^ mutants (J). In *ptc* mutants more PR precursors are formed compared to wildtype control. Analyzing the subtype identity of these PR precursors, we found that the ratio of Svp- vs Sal-positive cells is the same in *ptc* mutants and in wildtype. Number of all PRs: wildtype vs *ptc*^*9*^ p<0.001, t = -14.768; Number of all Sal-positive cells: wildtype vs *ptc*^*9*^ p<0.001, t = -6.608; Number of all Svp-positive cells: wildtype vs *ptc*^*9*^ p<0.001, t = -14.428 (I). Ratio of Sal-positive cells: wildtype vs *ptc*^*9*^ p = 0.876, t = 0.887; Ratio of Svp-positive cells: wildtype vs *ptc*^*9*^ p = 0.967, t = -0.627 (J). n = 14 (wildtype), 6 (*ptc*^*9*^) (I, J). Data is shown as mean and error bars as standard deviation. Circles represent numbers or percentages of individual samples. *** p<0.001and ns = not significant (I, J). Scale bars represent 20 μm.

Since it remains unknown how Hh signaling affects PR subtype identity, we analyzed the expression of the early PR precursor markers Ato and Sens in *ptc* mutants (Fig 4A, 4B, 4C and 4D). In agreement with previous observations, we found an increased number of Ato-expressing cells (Fig 4A and 4B) [6]. As expected from the expansion of Ato expression, we also observed an increased number of Sens expressing cells in *ptc* mutants, suggesting an increase of primary PR precursors (Fig 4C and 4D) [6,20]. By comparing the expression of the primary PR precursor marker Sal and the secondary PR precursor marker Svp in *ptc* mutants, we observed a significant increase of both precursor types (Figs 4E–4I and S4). The increase of PR precursors in *ptc* mutants is presumably due to an increase in cell number within the entire optic placode at stage 11. As a consequence more mature larval PRs were observed at the end of embryogenesis (Figs 4I, 4J and S3). Intriguingly, the ratio of Sal- and Svp-positive PRs in *ptc* mutants remained the same as in wildtype (Fig 4J).

**Fig 5:**
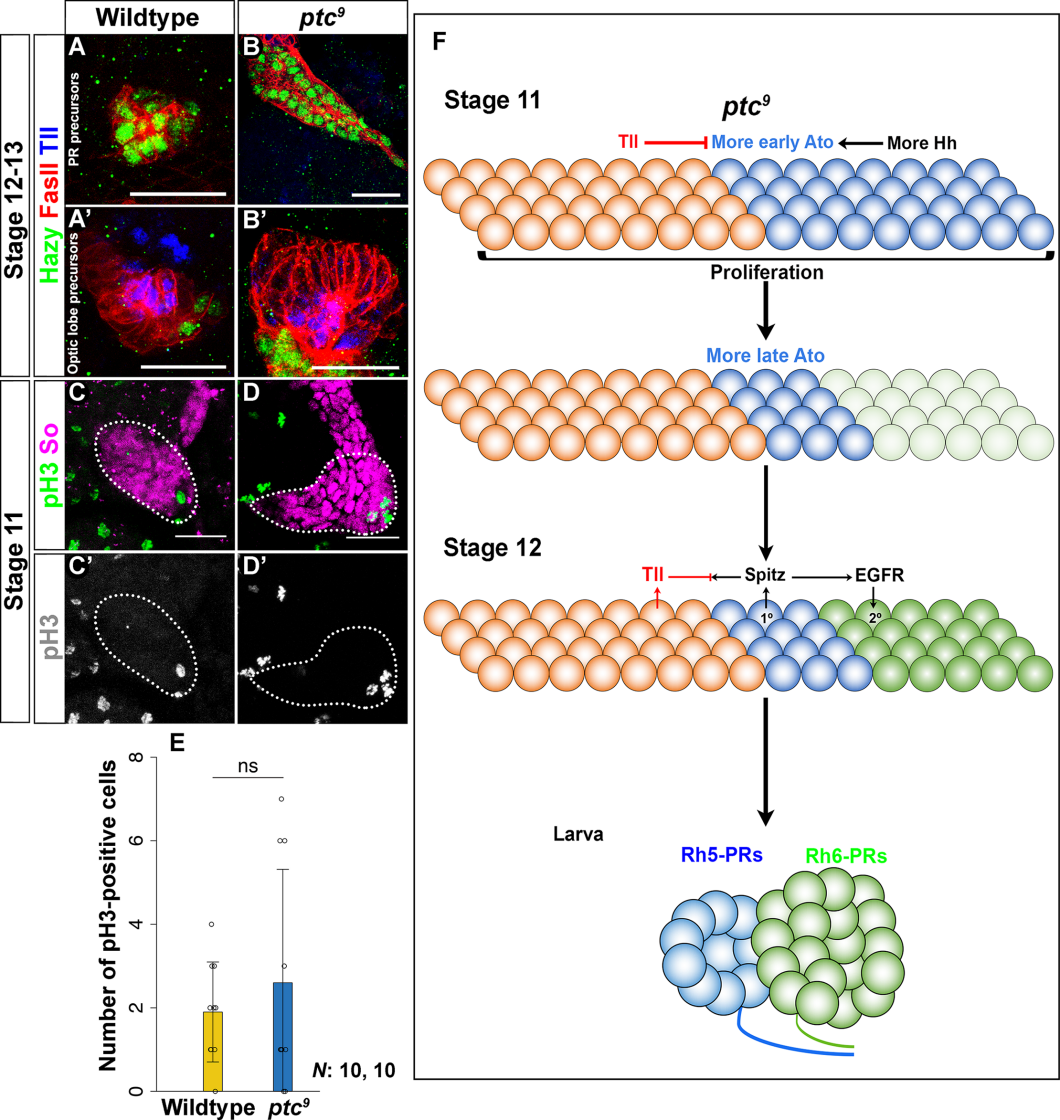
Hh signaling controls cell number in the optic placode. Tll (blue) expression in wildtype and *ptc*^*9*^ mutants (stage 12–13 embryos). Hazy (green) marks all PR precursors whereas FasII (red) labels larval eye and optic lobe primordium. (A, B) In the larval eye precursors, Tll is neither expressed in wildtype (A) nor in *ptc*^*9*^ mutants (B). Tll is expressed in the optic lobe precursors in wildtype (A’) and its expression is unchanged in *ptc*^*9*^ mutant embryos (B’). (C, C’, D, D’, E) Analysis of cell proliferation at stage 11 in the optic placode in wildtype and *ptc*^*9*^ mutant embryos by staining with anti-pH3 (green) antibody. So (red) was used to mark the area of the optic placode, from where pH3-positive cells were counted (white outline in C and D). The number of pH3-positive cells in the Eya-positive domain of *ptc*^*9*^ mutants is not significantly different from that of wildtype p = 0.4655, t(18) = -0.7457 (E). n = 10 (wildtype), 10 (*ptc*^*9*^) (E). Data is shown as mean and error bars as standard deviation. Circles represent numbers of individual samples. ns = not significant (E). (F) Schematic representation of Hh mediated optic placode patterning and acquisition of PR versus non-PR cell fate in the embryo. Hh signaling promotes Ato expression and Hh gain-of-function (in *ptc*^*9*^ mutants) show an increased number of Ato expressing cells in the optic placode [6]. Scale bars represent 20 μm.

Since Ato-dependent primary PR precursor specification is regulated both by Tll and Hh, we investigated whether Hh regulates Tll to control the formation of primary PR precursors. For this, we analyzed *tll* expression in the optic placode of *ptc* mutants and found that it is comparable to control embryos. *tll* is neither expressed in wildtype nor in *ptc* mutant PR precursors (Fig 5A and 5B), while it is expressed in a subset of wildtype or *ptc* mutant optic lobe precursors (Fig 5A’ and 5B’). Therefore, Tll and Hh might act in parallel to regulate the specification of primary PR precursors in the embryo.

Since Hh signaling controls cellular growth by regulating cell cycle specific genes during *Drosophila* compound eye development [23], we next investigated if the supernumerary PR precursors found in *ptc* mutants are a result of a general increase in cell number of the optic placode. To label proliferating cells in the optic placode, we marked mitotically active cells with the phospho-Histone H3 (pH3) antibody (Fig 5C and 5D) [24, 25]. However, we found no statistically significant change in the numbers of pH3-positive cells between wildtype and *ptc* mutants at stage 11 (Fig 5E).

Although the number of pH3-positive cells was not significantly increased at stage 11, the total number of cells in the optic placode of *ptc* mutants is significantly higher than in wildtype (Figs 4B, 4I and S3). These findings suggest that Hh signaling is required to control cell number in the optic placode and the larval eye probably by controlling proliferation or apoptosis (Fig 5F). Comparable to *tll* mutants, the ratio of primary to secondary PR precursors remains unchanged suggesting that the recruitment process of secondary PR precursors via EGFR signaling remains unaffected.

### A dual role of the Notch signaling pathway in optic placode patterning and larval PR specification

**Fig 6:**
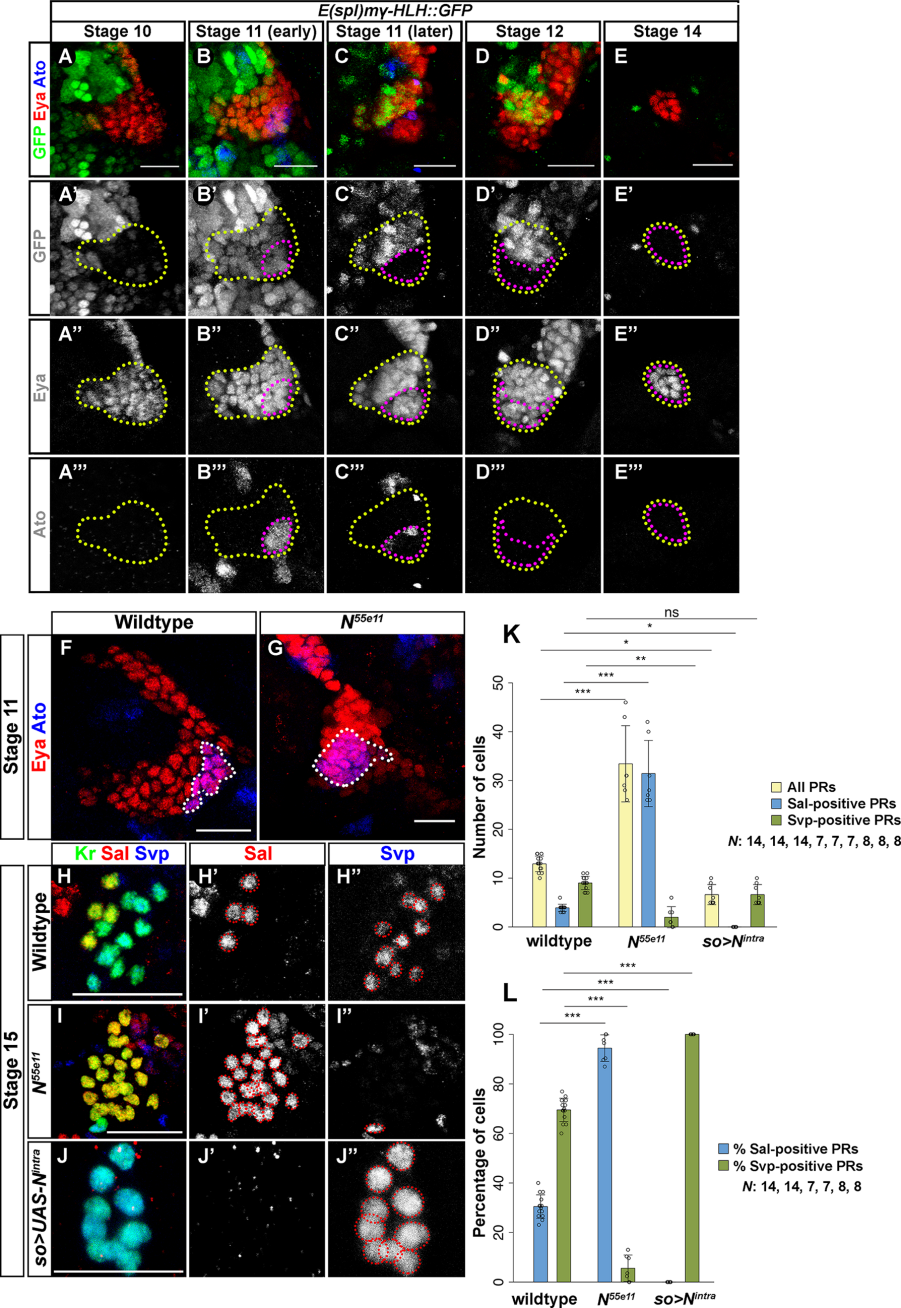
Notch regulates Ato-dependent PR cell fate in the embryo. (A-E) Notch activity in the optic placode at stages 10–14 determined by using the *E(spl)mγ-HLH*::*GFP* reporter line and staining embryos with anti-GFP (green), anti-Eya (red) and anti-Ato (blue) antibodies. Notch activity is dynamic: it is initially expressed in most cells in the placode early during stage 11 (outlined in yellow), and then it becomes excluded from the patch of Ato-expressing cells, which later will develop as PR precursors (outlined in purple). (F, G) Ato (blue, outlined in white) expression in the optic placode in wildtype and *N*^*55e11*^ mutant stage 11 embryos. The number of Ato expressing cells is significantly increased in *N*^*55e11*^ mutants (G). Sal (red) and Svp (blue) expression in the PR precursors at embryonic stage 15 in wildtype (H, H’, H”), *N*^*55e11*^ (I, I’, I”) and in the activated *Notch* overexpression (*so>UAS-N*^*intra*^) (J, J’, J”). Kr (green) marks all PR precursors at this stage. Four Sal expressing primary PR precursors (H’) and 8–10 Svp expressing secondary PR precursors (H”) are seen in wildtype. In *N*^*55e11*^ mutants the number of Sal expressing PRs is increased (I’), whereas in *N*^*intra*^ overexpression embryos they are absent (J’). In *N*^*55e11*^ mutants Svp expressing PRs are significantly reduced or absent (I”) while *N*^*intra*^ overexpression does not affect the number of Svp expressing PRs (J”). (K, L) Quantification of PR number (K) and percentage of Sal- and Svp-positive PRs (L) in wildtype, *N*^*55e11*^ mutant and *so>UAS-N*^*intra*^ overexpression embryos. In *N*^*55e11*^ mutants a higher number of PR precursors is specified compared to wildtype control (counted at stage 14–16). *N*^*55e11*^ mutants possess more Sal-positive primary PR precursors than wildtype, whereas no Sal-positive cells were found in *N*^*intra*^ overexpressing embryos. Conversely, Svp-positive secondary PR precursors are severely reduced in *N*^*55e11*^ mutants whereas in *N*^*intra*^ overexpressing embryos they represent 100% of the PR precursors. Number of all PRs: wildtype vs *N*^*55e11*^ p<0.001, t = -8.203; wildtype vs *so>N*^*Intra*^ p = 0.048, t = 2.634; Number of all Sal-positive cells: wildtype vs *N*^*55e11*^ p<0.001, t = -19.020; wildtype vs *so>N*^*Intra*^ p = 0.0312, t = 2.838; Number of all Svp-positive cells: wildtype vs *N*^*55e11*^ p = 0.0028, t = 3.692; wildtype vs *so*>*N*^*Intra*^ p = 0.6041, t = 1.308 (K). Ratio of Sal-positive cells: wildtype vs *N*^*55e11*^ p<0.001, t = -17.594; wildtype vs *so>N*^*Intra*^ p<0.001, t = 8.764; Ratio of Svp-positive cells: wildtype vs *N*^*55e11*^ p<0.001, t = 12.425; wildtype vs *so>N*^*Intra*^ p<0.001, t = -6.189 (L). n = 14 (wildtype), 7 (*N*^*55e11*^), 8 (*so*>*N*^*Intra*^) (K, L). Data is shown as mean and error bars as standard deviation. Circles represent numbers or percentages of individual samples. *** p<0.001, **p<0.01, *p<0.05 and ns = not significant (K, L). Scale bars represent 20 μm.

During optic placode development Notch has been implicated in maintaining neuroepithelial identity by suppressing the PR cell fate [10,26, 27]. Since the *Notch* mutant phenotype shows an increase in larval PR precursors [10], we first investigated how PR subtype specification is affected. When analyzing the expression of the *Notch* activity reporter *E(spl)mγ-HLH*::*GFP* in the optic placode we observed that at early stage 11, GFP expression was broadly distributed in the Eya-positive domain of the optic placode (Fig 6A and 6B). However, later, *E(spl)mγ-HLH*::*GFP* expression was excluded from the larval eye primordium, marked by Ato (Fig 6C–6E). To address how Notch may be involved in placode patterning, we next analyzed Ato expression in *Notch* loss-of-function mutants. We observed an expansion of the Ato-expressing domain as compared to wildtype embryos (Fig 6F and 6G). This increase in Ato-expressing cells, which give rise to PR precursors, indeed correlates with the supernumerary PRs found during later embryonic stages. Moreover, these supernumerary PRs expressed the transcription factors Hazy and Kr [17, 18], indicating that these cells are correctly specified (S2 Fig). Interestingly, at stage 11 the *Notch* mutant optic placode has as many cells as that of wildtype (an average of 46 cells), but it produces about twice the number of PRs (an average of 33) (Figs 6K, 6L and S3). To investigate PR subtype identity we analyzed Sal and Svp expression in *Notch* mutants. In agreement with previous observations we found a large increase in the total number of larval PR precursors (Fig 6H and 6I) [10]. This increase is based on the expansion in the number of Sal-positive cells (Fig 6H' and 6I') whereas no or a very reduced number of Svp-expressing cells was found in *Notch* mutants (Fig 6H'' and 6I''). This finding suggests that during PR development Notch regulates binary cell fate decision to promote secondary PR precursor identity while repressing primary PR precursor cell fate. Quantification of the number of Sal- and Svp-positive PRs in the *Notch* mutants also shows an increase of primary PR precursor and a decrease in secondary PR precursor cell numbers (Fig 6K and 6L).

Since Notch regulates larval PR subtype specification, we next investigated whether Notch is also sufficient to repress primary PR precursor cell fate in the embryo. We therefore ectopically expressed the intracellular domain of *Notch* (*N*^*intra*^*; active form*), derived by proteolytic cleavage of Notch protein [28], under the control of *so-Gal4*. We indeed found that ectopic expression of *N*^*intra*^ leads to a complete loss of Sal expression while all cells express the secondary PR precursor marker Svp (Fig 6J).

**Fig 7:**
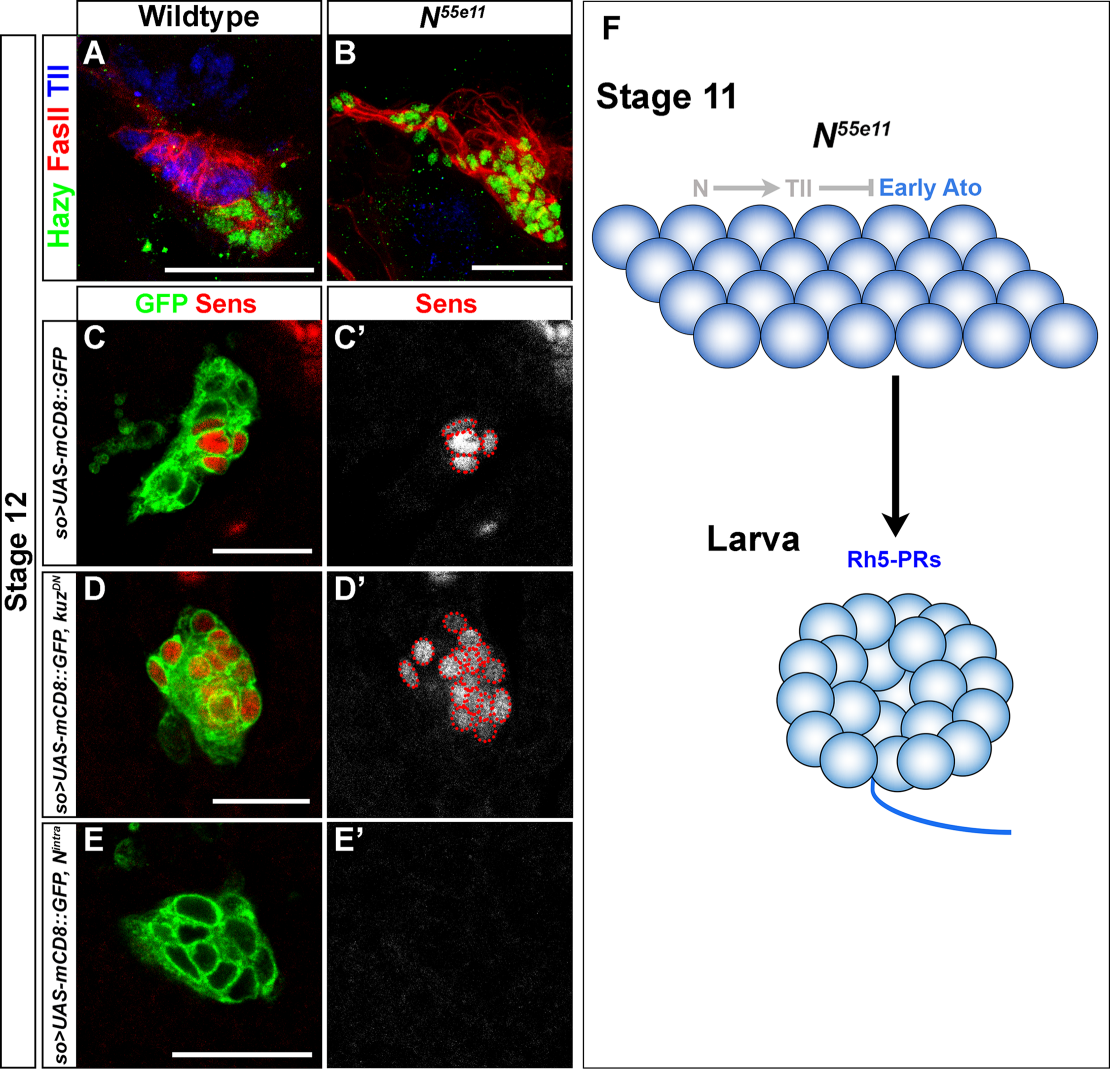
Notch represses sens-mediated binary cell fate decision and controls primary versus secondary PR precursor cell fate. (A, B) Tll (blue) expression analysis in the optic placode in wildtype and *N*^*55e11*^ mutant stage 12 embryos. Hazy (green) marks all PR precursors and FasII (red) labels larval eye and optic lobe primordium at this stage. Tll is expressed in the optic lobe primordium but not in the PR precursors in wildtype (A). In *N*^*55e11*^ mutant embryos Tll expression is completely absent (B). (C, C’ D, D’, E, E’) Sens (red) expression in wildtype and in embryos overexpressing a dominant negative form of Kuzbanian (*Kuz*^*DN*^) or the intracellular domain of Notch (*N*^*intra*^) in the optic placode by using a *so-Gal4*, *UAS-mCD8*::*GFP* recombinant transgenic line. Sens is normally expressed in four PR precursors in wildtype (C’), which adopt the primary PR precursor cell fate. Overexpression of *Kuz*^*DN*^ in the optic placode leads to an increase in the number of Sens expressing cells (D’), whereas overexpressing *N*^*intra*^ results in loss of Sens expression from the presumptive primary PR precursors (E’); z-projections of confocal sections. (F) Schematic representation of Notch mediated neuroepithelial patterning and regulation of cell fates in the embryonic optic placode. Notch activity is required for *tll* expression, and thus, indirectly and negatively regulates *ato* expression. Notch also represses *sens* expression. In *N*^*55e11*^ mutant this repression is removed and ectopic Sens induces primary PR precursor cell fate while repressing secondary PR precursor cell fate. Scale bars represent 20 μm.

We have previously shown that Sens is an additional marker for primary PR precursors [13]. We next investigated whether Notch regulates Sens activity and thereby controls PR subtype specification in the embryo. For this, we overexpressed a dominant-negative form of Kuzbanian (Kuz) (Kuz encodes a putative zinc metalloprotease responsible for proteolytic cleavage and release of the active form of Notch) [29] in the optic placode by using *so-Gal4*. Overexpression of *kuz*^*DN*^ results in an increase of Sens-positive cells (Fig 7C, 7C’, 7D and 7D’), suggesting that Notch acts upstream of Sens and regulates PR subtype specification by repressing Sens activity. To further support this notion, we next overexpressed the active form of Notch (*N*^*intra*^*)* in the optic placode by using *so-Gal4*. We indeed found that embryos having ectopic *N*^*intra*^ show a complete loss of Sens expression (Fig 7E and 7E’).

Since both Tll activity and Notch signaling regulate primary PR precursor specification, we next investigated whether *Notch* is required for *tll* expression. Indeed, we found that *tll* expression is completely lost in the optic placode in *Notch* mutant embryos (Fig 7A and 7B), suggesting that *Notch* acts genetically upstream of *tll*. We therefore attempted to rescue the *Notch* mutant phenotype by inducing expression of UAS-*tll* with *so-Gal4* while concomitantly blocking Notch signaling using UAS-*kuz*^*DN*^. Interestingly, in *so-Gal4/*UAS-*kuz*^*DN*^,UAS-*tll* the larval eye does not develop suggesting that pan-placodal expression of Tll is sufficient to repress photoreceptor precursor induction and further supporting the notion that Tll is acting downstream of Notch signaling (S5 Fig). In order to test a possible genetic interaction between the Notch and Hh signaling pathways we analyzed the expression of a *ptc-lacZ* reporter in the optic placode of *Notch* mutants. In this case, we still detected β-Gal expression in the respective neureopithelial domain, suggesting that these pathways may act in parallel (S6 Fig).

Our findings support a model in which Notch has two temporally distinct roles during larval eye formation. First, Notch signaling maintains neuroepithelial cells of the presumptive optic lobe by repressing *ato* expression in a Tll-dependent fashion (Fig 7F). Second, Notch represses the Sens mediated binary cell fate decision in the presumptive larval eye where it promotes secondary PR cell fate specification (Fig 7F).

## Discussion

### Spatial neuroepithelial and temporal patterning controls PR versus epidermal cell fate in the optic placode

Neurogenic placodes are transient structures that are formed by epithelial thickenings of the embryonic ectoderm and give rise to most neurons and other components of the sensory nervous system [30–34]. In vertebrates, cranial placodes form essential components of the sensory organs and generate neuronal diversity in the peripheral nervous system [35–37]. How neuronal diversity is generated varies from system to system, and different gene regulatory networks have been proposed for each particular type of neuron [17, 18, 38–40]. Interestingly, some transcription factors, like Atonal, play an evolutionary conserved role during neurogenesis both in *Drosophila* and in vertebrates [41].

**Fig 8:**
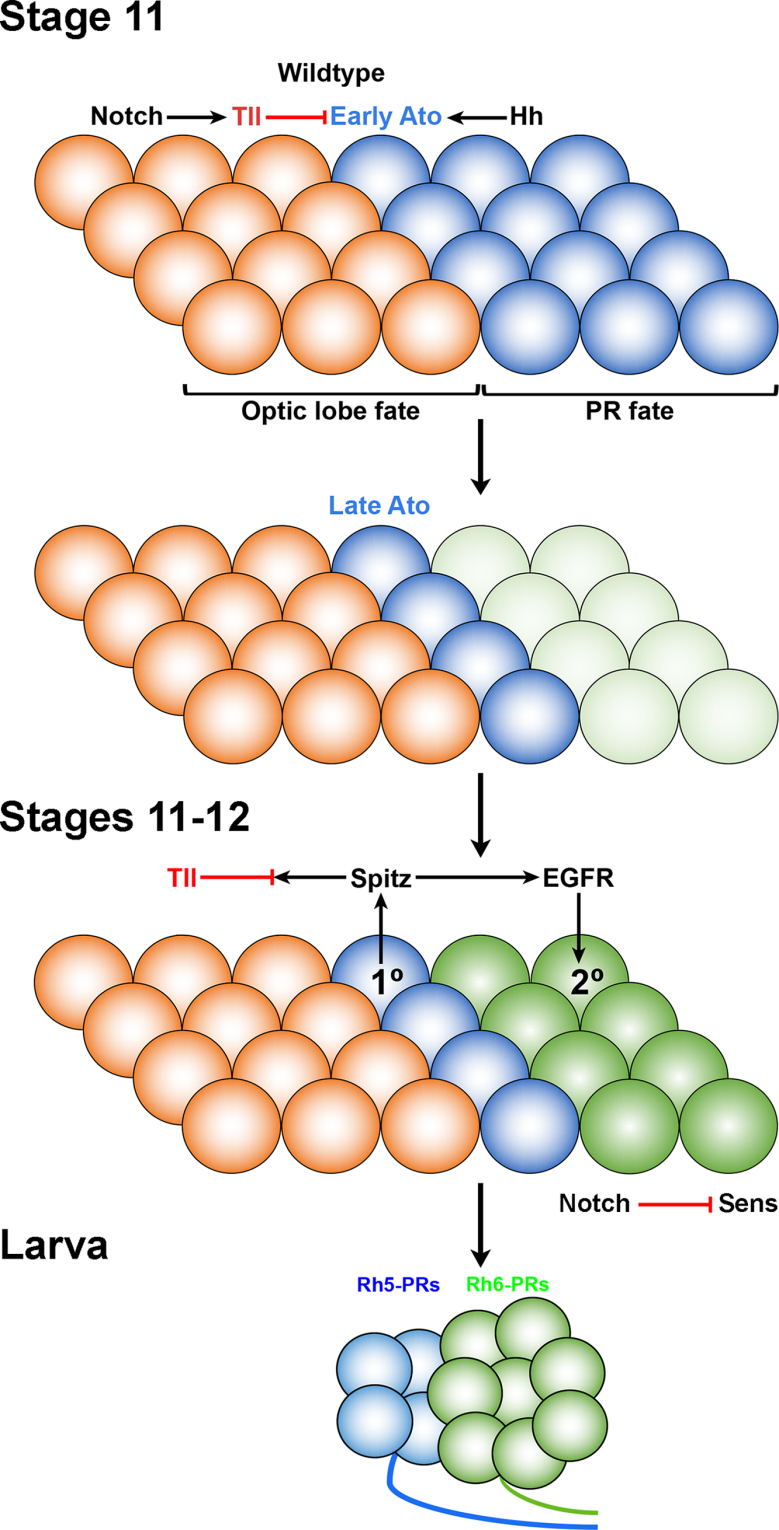
Model of neurogenic optic placode patterning and control of cell fate choices in the wildtype embryo. Schematic representation of neuroepithelial patterning and regulation of optic lobe neuroepithelium versus PR cell fate in the optic placode in *Drosophila*. Regulation of *ato* and *tll* expression by Hh and Notch signaling in the embryo is required for the acquisition of cell fate identity in the primordium of optic lobe and larval eye. Temporal expression of *ato* (early and late *ato*) in the larval eye primordium marks PR cell fate whereas *tll* expression in the optic lobe primordium marks optic lobe neuroepithelial cell fate. Tll represses *ato* expression and maintains a balance between PR and non-PR cells in the optic placode. Hh signaling promotes Ato-dependent primary PR precursor specification and controls cell number in the optic placode possibly by regulating cell proliferation. Notch activity maintains optic lobe neuroepithelial cell fate in the optic lobe by indirectly repressing *ato* activity in a Tll-dependent manner. In addition, at later stages Notch represses Sens and thereby promotes secondary PR precursor formation at the expense of primary PR precursors.

Neuroepithelial patterning of the *Drosophila* optic placode exhibits unique segregation of larval eye and optic lobe precursors during embryogenesis [4,6,20]. We identified genetic mechanisms that control early and late steps in specifying PR versus non-PR cell fate that ensure the expression of precursor cell fate determinants. During germband extension at stage 10, transcriptional regulators (*so*, *eya*, *ato and tll*) show complex and partially overlapping expression patterns in the optic placode. Their interactions with the Notch and Hh signaling pathways define distinct PR and non-PR domains of the larval eye and optic lobe primordium. Intriguingly, our results show a spatial organization of distinct precursor domains, supporting a new model of how the subdivision of precursor domains emerges. In agreement with previous studies initially the entire posterior ventral tip expresses Ato, defining the population of cells that give rise to PR precursors, while neuroepithelial precursors for the presumptive optic lobe are defined by Tll-expression in the anterior domain of the optic placode [4]. Subsequently, Ato expression ceases in the ventral most cells and thus gets restricted to about four primary PR precursors that are located directly adjacent to the Tll expression domain. Hence, a few cell rows are between the primary PR precursors and the ventral most edge of the optic placode. This is in agreement with a recent observation on the transcriptional regulation of *ato* during larval eye formation [42]. Thus, primary PR precursors are directly adjacent to the Tll-expressing cells while the Ato and Tll negative domain of secondary PR precursors is located at the posterior ventral most tip of the optic placode. Setting the Tll-Ato boundary is critical to define the number of putative secondary PR precursors, which can be recruited into the larval eye, probably via EGFR signaling [43]. We propose a model during which coordinated action of Hh, Notch and Tll restricts the initially broad expression of Ato to primary PR precursors (Fig 8). Lack of Tll results in a de-repression of Ato and results in an increased number of primary PR precursors, which in turn recruit secondary PR precursors. Interestingly, while *tll* mutants show an increase in both primary and secondary PR precursors, the ratio between both subtypes is maintained. This notion further displays similarities of ommatidal formation in the adult eye-antennal imaginal disc, where Ato expressing R8-precursors recruit R1-R6. In the eye-antennal disc, specification of R8-precursors determines the total number of ommatida and therefore also the total number of PRs, the ratio of R8 to outer PRs however always remains the same. Thus, the initial specification of primary PR precursors defines the total number of PRs in the larval eye similarly to R8 PRs, and the ratio of founder versus recruited cells remains constant. Interestingly, the maintenance of primary versus secondary PR precursor ratio is also maintained in *ptc* mutants further supporting this model.

### A dual role of Hh signaling in promoting PR-precursor identity and controlling cell number in the optic placode

During photoreceptor development in the eye-antennal imaginal disc *hh* is expressed in the posterior margin and is required for the initiation and progression of the morphogenetic furrow as well as the regulation of *ato* expression [19, 44, 45]. During embryogenesis the loss of *hh* results in a complete loss of the larval eye, while increasing Hh signaling (by means of mutating *ptc*) generates supernumerary PRs in the larval eye [6,20]. During early stages, we found an increase of Ato expression in *ptc* mutants suggesting that similarly to the eye-antennal disc Hh positively regulates *ato* expression. The observed increase of Ato-expressing cells is not due to a reduction of Tll but is likely due to increased cell proliferation in *ptc* mutants. Hh also controls proliferation during the formation of the *Drosophila* compound eye [46, 47].

### Two temporally distinct roles of Notch in promoting neuroepithelial cell fate and binary cell fate decision

During embryonic nervous system development Notch dependent lateral inhibition selects individual neuroectodermal cells to become neuroblasts. Notch represses neuroblast cell fate and promotes ectodermal cell fate [48–51]. During compound eye development, Notch regulates Ato expression and acts through lateral inhibition to select Ato expressing R8 PR precursors [52]. Similarly, during *Drosophila* larval eye development, Notch is required for regulating PR cell number by maintaining epithelial cell fate of the optic lobe primordium [20]. Inhibiting *Notch* signaling leads to a complete transformation of the optic placode to PRs of the larval eye [20]. In the absence of Notch signaling, Ato expression is expanded in the optic placode and as a result the total number of PRs is increased. Despite the increase of the overall PR-number the number of secondary PR precursors is significantly decreased or lost in the absence of Notch activity. In the compound eye Notch promotes R7 cell fate by repressing the R8-specific transcription factor Sens [53]. It was also proposed that genetic interaction between Notch and Sens is required for sensory organ precursor (SOP) selection in the proneural field in a spatio-temporal manner [54]. We found that during PR subtype specification Notch represses Sens expression, thereby controlling the binary cell fate decision of primary versus secondary PR precursors. Therefore, in the absence of Notch signaling, Sens expression represses the secondary PR precursor fate. As a result, all PR precursors are transformed and acquire primary PR precursor identity. In conclusion, we observed that Notch is essential for two aspects during optic placode patterning. First, Notch activity is critical for balancing neuroepithelial versus PR cell fate mediated through Tll-regulated Ato expression. Second, Notch regulates the binary cell fate decision of primary versus secondary PR precursor cell fate through the regulation of Sens expression.

## Material and methods

### Fly stocks

Flies were grown on standard food medium at 25°C and 12hr/12hr light dark cycle. Wildtype Canton S was used as controls in all cases. The following mutants and transgenic fly lines were used: *tll*^*49l*^ [55], *ptc*^*9*^ [56] and *N*^*55e11*^ [57] mutant lines were used to analyze Tll, Hh and Notch function, respectively. *tll*::*GFP* [58] and *E(spl)mγ-HLH*::*GFP* transgenic lines were used to analyze Tll expression and Notch activity pattern. *UAS-tll* (gifts from M. Kurusu) [59], *UAS-N*^*intra*^ [60] and *UAS-kuz*^*DN*^ [61] transgenic lines were used to overexpress Tll, the Notch intracellular domain and the Kuzbanian dominant negative form. *so-Gal4* [62] was used to overexpress genes early in the optic placode and *UAS-mCD8*::*GFP* [63] was used to mark Gal4 expression pattern in the embryo. We also used *ato*^*w*^ [64] (kindly provided by B. Hassan) and *ptc-lacZ* (gift of K. Basler).

### Generation of So antibody and immunohistochemistry

For So antibody production, the complete *so* coding sequence from the cDNA clone FI01103 was PCR amplified and cloned into pGEX-6P-1 in frame with the GST-Tag. So protein was expressed in BL-21 cells and purified with Glutathione-sepharose beads (GE Healthcare) cleaving it from the GST-tag at the HRV 3C site (THERMO Scientific). The purified protein was sent to Eurogentec for immunization of two guinea pigs.

For immunohistochemistry, *Drosophila* embryos were dechorionated, fixed and immunostained as described in [13]. Briefly, embryos were collected after keeping flies on apple juice plates for overnight at 25°C. Embryos were dechorionated by using 50% bleach solution for 7 minutes. Embryos were fixed for 25–30 minutes by using equal volumes of n-heptane and 4% formaldehyde (made in 1XPBS; Phosphate buffered saline) solutions. They were devitellinized and stored in methanol at -20°C. Immunostaining of embryos were initiated by washing them in 1xPBS containing 0.3% Triton X-100 (PBST) three times for 30 minutes each. Primary antibody dilutions were made in PBST and embryos were incubated in the primary antibody for overnight at 4°C. On the next day, three washes were performed using PBST for 30 minutes each. Secondary antibodies were diluted in PBST and embryos were incubated with the secondary antibody solution for two hours at room temperature or overnight at 4°C. Washing was performed again and samples were mounted by using Vectashield H-1000 (Vector laboratories). The following primary antibodies were used: mouse anti-Eya and mouse anti-FasII (both at 1:20; Developmental Studies Hybridoma Bank), guinea pig anti-So (1:100; this work), rabbit anti Hazy (1:500) [65] rabbit anti-Ato (1:5000) [66], chicken anti-GFP (1:2000; Life technologies), guinea pig anti-Kr (1:200) and guinea pig anti-Tll (1:100; gifts from J. Jaeger), rabbit anti-Rhodopsin 6 (1:10000) [67], rabbit anti-Rhodopsin 5 (1:40) [68], rabbit anti-Sal (1:300) [69], mouse anti-Svp (1:100) [70], guinea pig anti-Sens (1:800) [71], rabbit anti-Hazy (1:500) [65] and rabbit anti-pH3 (1:200) (Upstate Biotechnology). The following secondary antibodies were used: Alexa488, Alexa568 and Alexa647 (1:200; Molecular Probes).

### Quantification of cell number and percentage of PRs

For cell quantifications in wildtype and mutants, we calculated the mean for each category and determined the standard deviation. Circles represent numbers or percentages of individual samples. Standard functions in MATLAB (MathWorks) (“ttest2”) and in RStudio (RStudio, Inc.) (“aov” and “glht(multcomp)”) were applied for statistical analysis.

A one-way ANOVA followed by Dunnett’s test was applied to test the number of all PRs, the number of Sal-positive PRs, the number of Svp-positive PRs and the number of all optic placode cells of the different genotypes respectively against the particular numbers of the wildtype control.

A one-way ANOVA followed by Dunnett’s test was performed to test the percentage of Sal-positive PRs and the percentage of Svp-positive PRs of the different genotypes respectively against the specific values of the wildtype control.

A two-tailed two sample t test was applied to test the number of pH3-positive cells of wildtype control and *ptc*^*9*^ mutants against each other.

Rejection of the null hypothesis that the numbers or ratios of cells of data sets are the same: (***) means p-value<0.001, (**) means p-value<0.01 and (*) means p-value<0.05.

### Laser confocal microscopy and image processing

Confocal stacks were collected by using a 40x (NA 1.3) oil or a 63x (NA 1.3) glycerol immersion objective on a TCS Leica SP5 confocal microscope. Acquired image resolution was 1024x1024 pixels and optical sections were 1 to 1.5μm. Fiji/ImageJ was used for image analysis and Adobe Photoshop CS6 software was used for brightness/contrast adjustment and background subtraction. Figures were assembled in Adobe Photoshop CS6.

## Supporting information

S1 Fig pH3 expression in the optic placode. We analyzed the number of mitotically active cells in the optic placode by staining against pH3 (green). We also used antibodies against Eya (red) to identify the optic placode (yellow outline), and we counterstained with DAPI (grey). At stage 10 virtually all Eya-positive cells in the placode co-express pH3 (A), but very few express it later, during stages 11 (B) and 12 (C). Scale bars represent 20 μm.
(TIF)

S2 Fig The PR marker Hazy is expressed in all PR precursors in *tll*^*49l*^, *ptc*^*9*^, and *N*^*55e11*^ mutants. We stained against Kruppel (green) to label the larval eye in embryos around stage 14, and co-stained against Hazy (magenta). Hazy is a transcription factor that regulates the development of all types of *Drosophila* PRs in wildtype conditions [17, 18, 65]. Similar to wildtype, all PR precursors express Hazy in *tll* (A), *ptc* (B) and *Notch* (C) mutant embryos. Scale bars represent 20 μm.
(TIF)

S3 Fig Quantification of optic placode cell numbers. The optic placode contains the same number of cells in *N*^*55e11*^ mutants and so>tll embryos compared to wildtype embryos (counted at stage 11). The number of cells in the optic placode is increased in *tll*^*49I*^ mutants and *ptc*^*9*^ mutants compared to wildtype embryos (counted at stage 11). Number of all optic placode cells: Anova: p<0.001 F(4,43) = 15.05; wildtype vs *tll*^*49I*^ p<0.001, t = -5.627; wildtype vs *so>tll* p = 1, t = 0.057; wildtype vs *ptc*^*9*^ p<0.001, t = -4.738; wildtype vs *N*^*55e11*^ p = 0.997, t = -0.259. n = 11 (wildtype), 8 (*tll*^*49I*^), 10 (*so*>*tll*), 10 (*ptc*^*9*^), 9 (*N*^*55e11*^). Data is shown as mean and error bars as standard deviation. Circles represent numbers of individual samples. *** p<0.001 and ns = not significant.
(TIF)

S4 Fig Opsin expression in *ptc* mutants. We dissected the larval eyes of *ptc*^*9*^ embryos at stage 17, and stained them with antibodies against Rhodopsin 6 (green), Rhodopsin 5 (blue), and Elav (red). We found that the additional PRs that are formed in *ptc* mutants correctly expressed these terminal differentiation markers (A, B). Scale bars represent 20 μm.
(TIF)

S5 Fig Tll overexpression in *Notch* mutants. We attempted to rescue the Notch loss-of-function phenotype (*so>Kuz*^*DN*^) by overexpressing Tll. For this, we stained by using Kruppel (green, arrowhead) as a larval eye marker and counter-stained with DAPI (magenta). Under these conditions, we were able to identify the Bolwig's organ in control (A), but not in experimental embryos (B). Stage 16 embryos are shown. Scale bars represent 100 μm.
(TIF)

S6 Fig Ptc expression in *Notch* mutants. We stained *ptc-lacZ* embryos at stage 11 with antibodies against Eya (green, to label the optic placode) and βGal (magenta). The reporter was similarly expressed in the optic placode of both control (A) and *N*^*55e11*^ (B) mutant animals. Scale bars represent 20 μm.
(TIF)
