# Supplementary figures and images for: Patterning mechanisms diversify neuroepithelial domains in the Drosophila optic placode
Source: PLoS Genet. 2018 Apr 20;14(4):e1007353. doi: 10.1371/journal.pgen.1007353 (PMC5937791; doi:10.1371/journal.pgen.1007353)

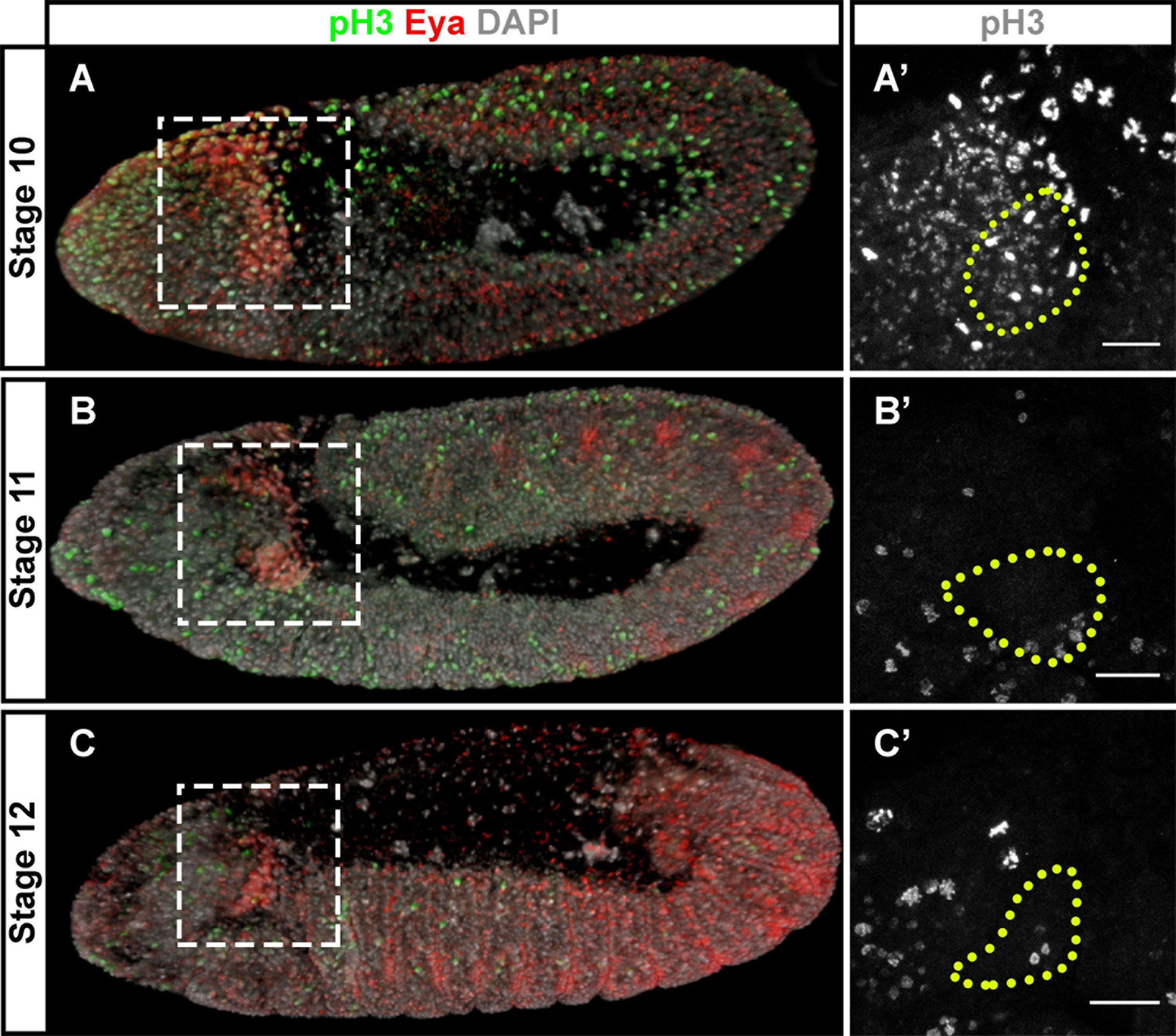

Supplement: S1 Fig — We analyzed the number of mitotically active cells in the optic placode by staining against pH3 (green). We also used antibodies against Eya (red) to identify the optic placode (yellow outline), and we counterstained with DAPI (grey). At stage 10 virtually all Eya-positive cells in the placode co-express pH3 (A), but very few express it later, during stages 11 (B) and 12 (C). Scale bars represent 20 μm. (TIF) [file pgen.1007353.s001.tif]

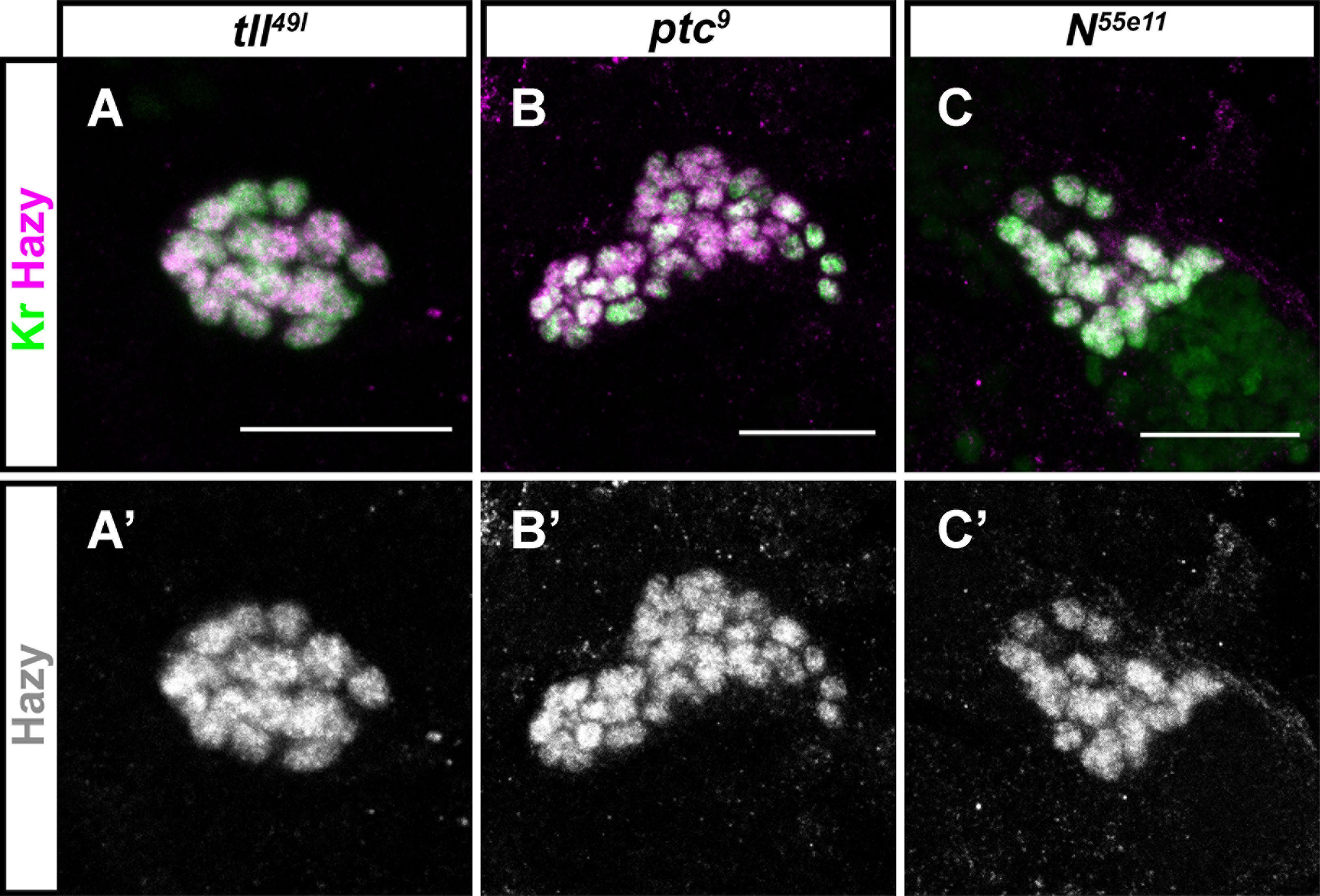

Supplement: S2 Fig — We stained against Kruppel (green) to label the larval eye in embryos around stage 14, and co-stained against Hazy (magenta). Hazy is a transcription factor that regulates the development of all types of Drosophila PRs in wildtype conditions [17, 18, 65]. Similar to wildtype, all PR precursors express Hazy in tll (A), ptc (B) and Notch (C) mutant embryos. Scale bars represent 20 μm. (TIF) [file pgen.1007353.s002.tif]

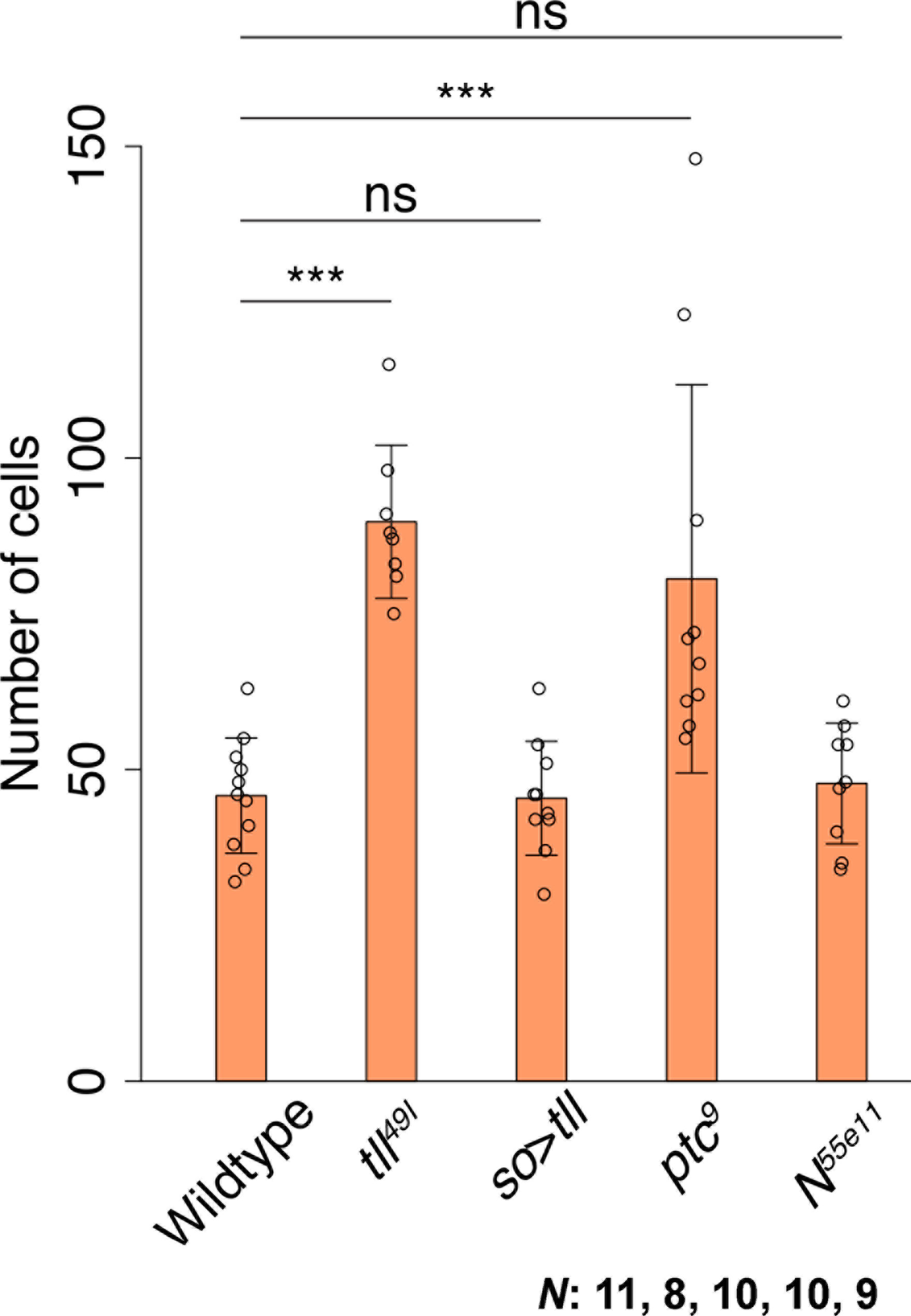

Supplement: S3 Fig — The optic placode contains the same number of cells in N55e11 mutants and so>tll embryos compared to wildtype embryos (counted at stage 11). The number of cells in the optic placode is increased in tll49I mutants and ptc9 mutants compared to wildtype embryos (counted at stage 11). Number of all optic placode cells: Anova: p<0.001 F(4,43) = 15.05; wildtype vs tll49I p<0.001, t = -5.627; wildtype vs so>tll p = 1, t = 0.057; wildtype vs ptc9 p<0.001, t = -4.738; wildtype vs N55e11 p = 0.997, t = -0.259. n = 11 (wildtype), 8 (tll49I), 10 (so>tll), 10 (ptc9), 9 (N55e11). Data is shown as mean and error bars as standard deviation. Circles represent numbers of individual samples. *** p<0.001 and ns = not significant. (TIF) [file pgen.1007353.s003.tif]

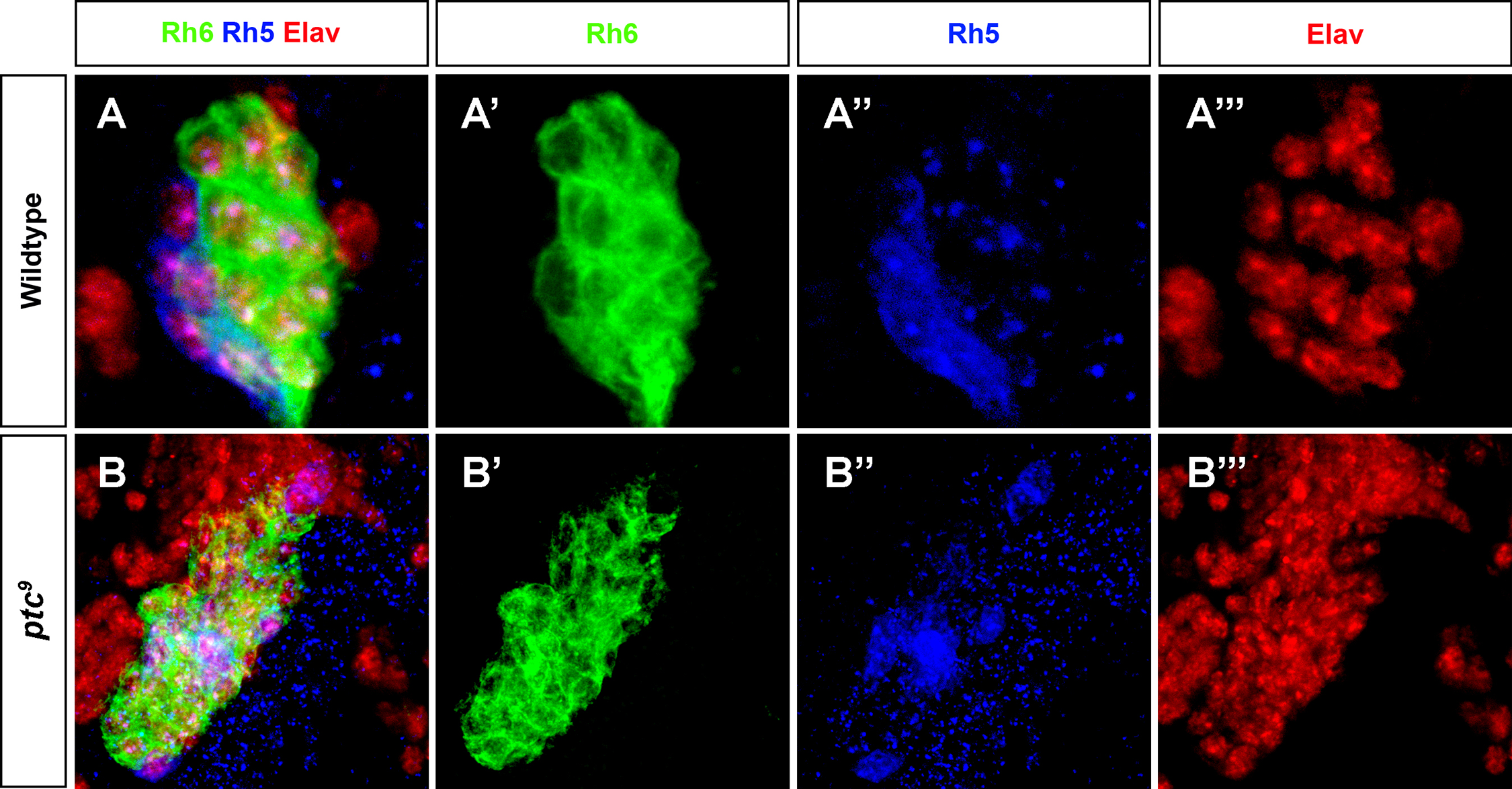

Supplement: S4 Fig — We dissected the larval eyes of ptc9 embryos at stage 17, and stained them with antibodies against Rhodopsin 6 (green), Rhodopsin 5 (blue), and Elav (red). We found that the additional PRs that are formed in ptc mutants correctly expressed these terminal differentiation markers (A, B). Scale bars represent 20 μm. (TIF) [file pgen.1007353.s004.tif]

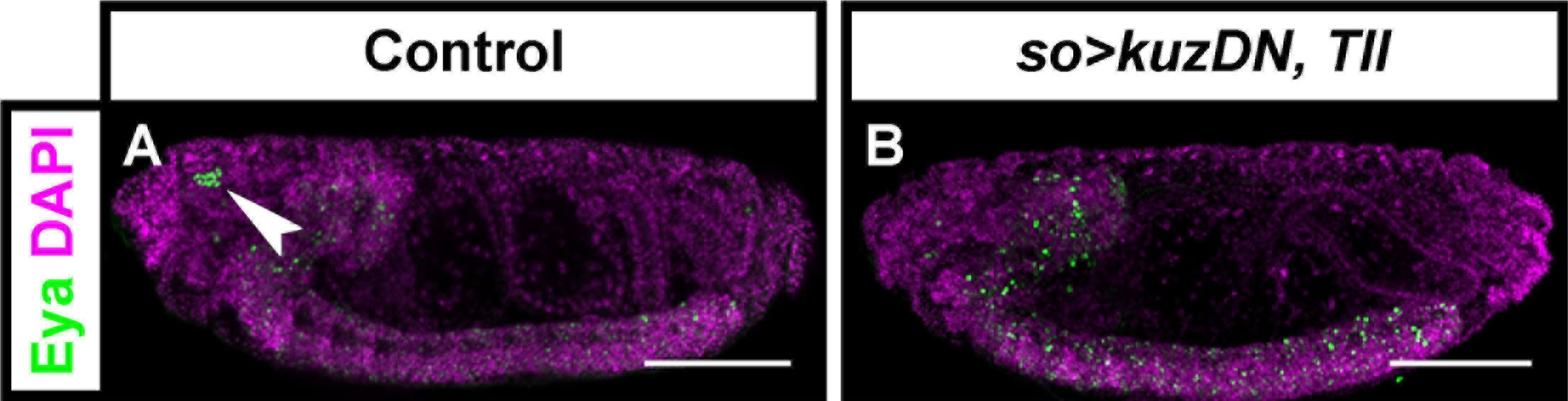

Supplement: S5 Fig — We attempted to rescue the Notch loss-of-function phenotype (so>KuzDN) by overexpressing Tll. For this, we stained by using Kruppel (green, arrowhead) as a larval eye marker and counter-stained with DAPI (magenta). Under these conditions, we were able to identify the Bolwig's organ in control (A), but not in experimental embryos (B). Stage 16 embryos are shown. Scale bars represent 100 μm. (TIF) [file pgen.1007353.s005.tif]

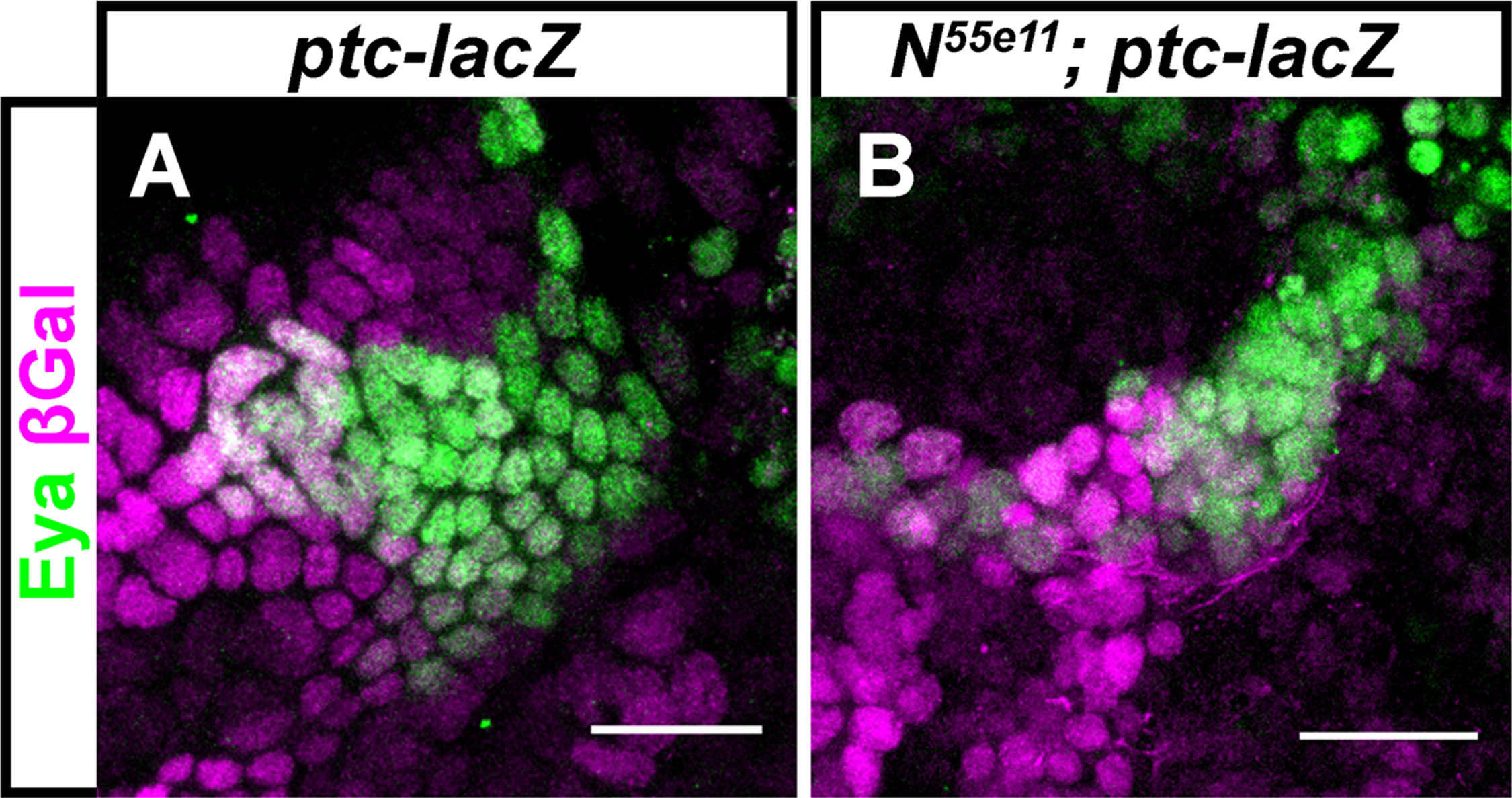

Supplement: S6 Fig — We stained ptc-lacZ embryos at stage 11 with antibodies against Eya (green, to label the optic placode) and βGal (magenta). The reporter was similarly expressed in the optic placode of both control (A) and N55e11 (B) mutant animals. Scale bars represent 20 μm. (TIF) [file pgen.1007353.s006.tif]
